# Assessment of Genetic Diversity of Bermudagrass (*Cynodon dactylon*) Using ISSR Markers

**DOI:** 10.3390/ijms13010383

**Published:** 2011-12-29

**Authors:** Tayebeh Mohammadi Farsani, Nematollah Etemadi, Badraldin Ebrahim Sayed-Tabatabaei, Majid Talebi

**Affiliations:** 1 Department of Agricultural Biotechnology, College of Agriculture, Isfahan University of Technology, Isfahan 84156-83111, Iran; E-Mails: Taiebeh_farsani@yahoo.com (T.M.F.); sayedt@cc.iut.ac.ir (B.E.S.-T.); 2 Department of Horticultural Science, College of Agriculture, Isfahan University of Technology, Isfahan 84156-83111, Iran; E-Mail: etemadin@cc.iut.ac.ir

**Keywords:** bermudagrass, genetic diversity, ISSR, Iran

## Abstract

Bermudagrass *(Cynodon* spp.) is a major turfgrass for home lawns, public parks, golf courses and sport fields and is known to have originated in the Middle East. Morphological and physiological characteristics are not sufficient to differentiate some bermudagrass genotypes because the differences between them are often subtle and subjected to environmental influences. In this study, twenty seven bermudagrass accessions and introductions, mostly from different parts of Iran, were assayed by inter-simple sequence repeat (ISSR) markers to differentiate and explore their genetic relationships. Fourteen ISSR primers amplified 389 fragments of which 313 (80.5%) were polymorphic. The average polymorphism information content (PIC) was 0.328, which shows that the majority of primers are informative. Cluster analysis using the un-weighted paired group method with arithmetic average (UPGMA) method and Jaccard’s similarity coefficient (*r* = 0.828) grouped the accessions into six main clusters according to some degree to geographical origin, their chromosome number and some morphological characteristics. It can be concluded that there exists a wide genetic base of bermudograss in Iran and that ISSR markers are effective in determining genetic diversity and relationships among them.

## 1. Introduction

*Cynodon dactylon* (L.) Pers. (common bermudagrass) is the most important member of the genus *Cynodon* because of its widespread distribution in warmer parts of the world and its use as livestock herbage and turf [1]. Harlan and de Wet describe the taxon as the ubiquitous, cosmopolitan weed of the world, containing an enormous variation ranging from small, fine turfgrasses used as golf course putting green turf to robust types grown for pasture or hay [2].

The center of diversity for some races of *Cynodon dactylon* (L.) Pers. was reported to be Turkey, Iran, Afghanistan and the western part of Pakistan, which could be potentially rich in the desirable genes and genotypes [1,2]. Characterizing its genetic diversity for germplasms is an essential step in selection and breeding of this grass.

The commonly used polymerase chain reaction (PCR) based DNA marker systems are random amplified polymorphic DNA (RAPD), amplified fragment length polymorphism (AFLP) and more recently simple sequence repeats (SSRs) or microsatellites [3]. The major limitations of these methods are low reproducibility of RAPD, high cost of AFLP and the need to know the flanking sequences to develop species specific primers for SSR polymorphism [4]. Markers such as inter-simple sequence repeat (ISSR) [5] are widely used in genetic diversity studies because they need no prior DNA sequence information, development costs are low, and laboratory procedures can easily be transferred to any plant species [6]. ISSR is a technique that overcomes most of these limitations [5,7]. This technology has been used to DNA fingerprint a wide range of crops [5,7,8–10] and to understand ploidy complex and the geographic origin of some plant species [11,12–16]. DNA profiling techniques that have been successfully used in assessing relatedness of *Cynodon* accessions includes DNA amplification fingerprinting (DAF) [17], RAPD [18,19], AFLP [20,21], ISSR [22], SSR [23] and chloroplast specific simple sequence repeat length polymorphism (CpSSRLP) [20].

In this study, we reported the feasibility of the ISSR-based PCR as an interesting approach in providing accurate molecular markers for investigating the genetic diversity among bermudagrass accessions.

## 2. Results

### 2.1. ISSR Amplification

**Figure 1:**
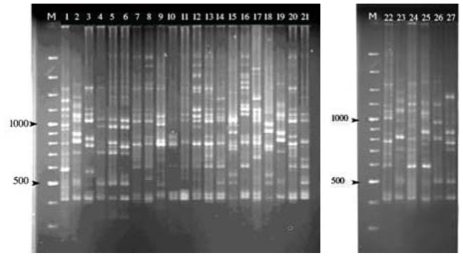
Bands profiles generated by the primer ISSR-7 with the sequence 5′-(GA)8T-3′, marker 100(M), Tifdwarf, Tifway, Tifgreen, Midlawn, 1-Lr, 3-Gs, 6-Gs, 11-Ns, 13-Ns, 15-Bs, 17-GN1, 18-Gr, 19-Gcr, 24-II, 27-II, 36-Tr, 39-II, 44-II, 51-Chg, 52-Chg, 53-Chr, 55-Chg, 56-Chr, 58-Chr, 59-Chf, 78-Ts, 88-Khl.

The fourteen selected ISSR primers produced 389 bands with an average of 27.78 bands per primer, of which 313 (80.5%) were polymorphic with an average of 22.36 bands per primer. The different ISSR primers amplified the number of bands from 20 (ISSR-14) to 40 (ISSR-11) having a range of 400 to 1200 bp fragments size. The number of polymorphic fragments detected by each primer ranged from 16 to 27. The most polymorphism was shown by ISSR-5, which showed 92.8% polymorphism. The mean of polymorphism information content (PIC) value for primers was 0.328 which ranged from 0.205 (ISSR-5) to 0.433 (ISSR-15) (Table 1). Figure 1 shows the banding pattern of genotypes generated by the primer ISSR-7.

### 2.2. Genetic Variation and Similarity among Genotypes

The average values of observed number of alleles (Na), effective number of alleles (Ne), Nei’s gene diversity (He) and Shannon’s information index (I) for all primers were 2, 1.56, 0.33 and 0.56, respectively.

The similarity matrix of the 27 genotypes from ISSR data using Jaccard’s coefficient showed that the genetic relationships of genotypes were different varying between 0.057 and 0.524 (data not shown). The lowest similarity was revealed between “18-Gr” and “27-II” genotypes (0.057), whereas the highest similarity was between “3-Gs” and “6-Gs” genotypes (0.524). The latest two common bermudagrass accessions, “3-Gs” and “6-Gs”, are tetraploid and are collected from the same origin (Table 2) with some similar morphological characteristics such as length and width of leaf and diameter of rhizome. The very close genetic relationship of these accessions suggests that they are a clonal propagule of a single plant.

### 2.3. Cluster Analysis

The data obtained from ISSR analysis of 27 bermudagrass genotypes was subjected to cluster analysis. In order to recognize the best clustering and similarity coefficient methods and the cophenetic correlation coefficient, a measure of the correlation between cophenetic matrix constructed from tree and similarity matrix, was calculated for each method combination. From different methods, the highest value (*r* = 0.828) was observed for the un-weighted paired group method with arithmetic average (UPGMA) clustering method based on Jaccard’s similarity coefficient, suggesting that the cluster analysis represents the similarity matrix.

**Figure 2:**
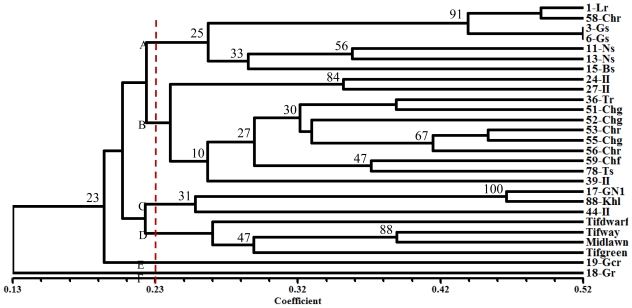
UPGMA (un-weighted paired group method with arithmetic average) dendrogram depicting patterns of genetic diversity for 23 *Cynodon dactylon* (L.) and Tifdwarf, Tifway, Tifgreen, Midlawn using 313 ISSR markers.

The UPGMA dendrogram grouped the 27 accessions into six clusters considered as A, B, C, D, E and F with a similarity coefficient of 0.23 (Figure 2). Seven tetraploid accessions were grouped together as cluster A of which three (“1-Lr”, “3-Gs”, “6-Gs”) and four (“11-Ns”, “13-Ns”, “15-Bs”, “58-Chr”) of them were from Charmahal Bakhtiari and Isfahan provinces, respectively. These accessions have some similar morphological characteristics such as leaf and rhizome color. Genetic similarity coefficient values for accessions in cluster A ranged from 0.198 to 0.524.

The major cluster B included the largest number of accessions (11 tetraploides) that had some similar morphological characteristics, such as leaf width, length of flower, stem and leaf, and stolon color. The grouping of these accessions from the same province (Isfahan) may reflect their having a common geographic origin. Genetic similarity coefficient values in this group were from 0.128 to 0.458.

Cluster C embraced two diploid accessions (“17-GN1” and “88-Khl”) and one triploid accession (“44-II”). These accessions have also similar morphological characteristics such as width of leaf and stolon color.

Four triploid hybrids (*Cynodon dactylon* × *Cynodon transvaalensis*), Tifgreen, Tifdwarf, Tifway and Midlawn were separated from all accessions and grouped together as cluster D.

Two tetraploid accessions, “19-Gcr” and “18-Gr”, collected from Gilan (Annual rainfall 1220 mm) were separated from all accessions in single clusters as labeled E and F, respectively in Figure 2.

Principal coordinate analysis (PCoA) based on genetic similarity metrics was used to visualize the genetic relationships among accessions. The first three eigenvectors accounted for 25.61% of the total molecular variation. Since the original data are not highly correlated in PCoA, the first few PCs do not explain much of the original variation. Assessment of genetic relationships on the basis of the first three PCs could lead to misleading interpretations and therefore analysis of genetic relationships among accessions should be based on cluster analysis as well as the optimal number of PCs that explain the maximum amount of the original data.

## 3. Discussion

The results obtained in our study showed that ISSR markers measured sufficient polymorphism for DNA typing and can be successfully used in bermudagrass germplasm characterization and for fingerprinting purposes. Similar results were obtained in several studies using other molecular markers such as RAPD [11,18].

The polymorphic information content (PIC) and the polymorphism rate (*P*) were used to measure the genetic diversity in bermudagrass accessions. High, medium or low polymorphism is in accordance with PIC > 0.5, 0.5 > PIC > 0.25 and PIC < 0.25, respectively [24,25]. Moreover, the mean value of the PIC obtained in this study was 0.328, indicating that the primers could develop medium polymorphism which is useful for genetic variation of genotypes studied in this research.

The average values of Na, Ne, He and I indicated that the background genetic data of germplasm accessions should be considered for integrated application in the breeding programs for germplasm improvement. It should be noted that, as a measure of genetic variation, the effective number of alleles (Ne) or expected heterozygosity (He) is more appropriate than the actual number of alleles (Na), since the latter depend on the sample size [26].

Bootstrapping was effectively utilized for estimating the statistical support to the internal branches of the tree. The internal tree branches that have >70% bootstrap are likely to be correct at the 95% level. However, a high bootstrap percentage still does not guarantee that long branch attractions have not biased the results. Also, in many cases, the overall tree structure provides better information than a particular branch. Wherever a priori information regarding genetic relationships is available, it is preferable to apply it in the interpretation [27]. Therefore, a priori information about accessions was used in branching the constructed tree.

Hybridization is one of the most common methods used to create variation in bermudagrass [28]. Most of the bermudagrass cultivars are interspecific hybrids between *Cynodon dactylon* and *Cynodon transvaalensis*. In this study, we included four hybrids, which were divided into two subgroups on the UPGMA tree. Among these hybrids, “Tifgreen” and its putative spontaneous somatic mutant (Tifdwraf) were placed in two separate subgroups, whereas, “Tifway” and “Midlawn” were placed in the same subgroup. Zhang *et al*. and Caetano-Anolles used AFLP and DAF techniques to effectively differentiate “Tifgreen” and the putative somatic mutants [29,30]. They showed that “Tifgreen” and “Tifdwarf” formed separate groups that are consistent with the results of this study. Triploidy forms were not indicated for *Cynodon dactylon* in the taxonomic revision of the genus as listed by Harlan *et al*. [1] probably because their collection contained none [11,31].

Cluster analysis was able to cluster bermudagrass accessions based on geographical origin, which is in agreement with previous observations of Li *et al*. in Chinese natural bermudagrass (*Cynodon dactylon*) germplasm using ISSR markers [22]. This showed that there was an incomplete direct relationship between the origin of the accessions and the molecular clusters. The reasons might be due to genetic overlap occurring in the bermudagrass accessions from two different regions, open pollination behavior of *Cynodon* plants and exchange of germplasm resources from different regions. This constructed dendrogram may be able to discriminate between genetically different chromosome numbers of *Cynodon dactylon* as reported by Anderson *et al*. [17], Etemadi *et al*. [18] and Gulsen *et al*. [11].

## 4. Experimental Section

### 4.1. Plant Materials and DNA Extraction

Twenty seven bermudagrass genotypes were included in this study. Among them, 23 genotypes were common bermudagrass types (*Cynodon dactylon*) and four were triploid hybrids of *Cynodon dactylon × Cynodon transvaalensis* (Tifdwarf, Tifgreen, Tifway and Midlawn). Stolons of twenty-three accessions of *Cynodon dactylon* (L.) Pers. had been collected from Isfahan, Charmahal Bakhtiari, Gilan and Mazandaran, provinces of Iran (Table 2). Each accession was grown in 20 cm diameter pots under green-house conditions. The green-house was maintained at 25 ± 1 °C. Bermudagrass DNA samples were isolated from fresh leaf tissue according to Dellaporta *et al*. [32].

### 4.2. ISSR Assays

Fourteen random ISSR primers (Operan technologies, Inc, Japan) were used to amplify DNA fragments of the selected materials. These primers were selected based on preliminary screening, producing a higher level of polymorphism and reproducible fragment patterns (Table 1). ISSR reactions were conducted in a volume of 25 μL consisting of 2.5 μL of 10× PCR buffer, 1.5 mM MgCl_2_, 1 U of *Taq* DNA polymerase (Roch Co. Germany), 200 μM of dNTPs, 1.25 μM of each primer, and template DNA of approximately 50 ng.

Amplifications were carried out in a Thermal Cycler (T-1 Theroblock, Germany) with the following PCR program: 3 min of initial denaturing at 94 °C, 40 cycles of 94 °C for 45 s, 1 min for annealing at the primer-specific melting temperature, and 72 °C for 4 min; followed by a final extension of 10 min at 72 °C. The amplified fragments were separated on a 1.5% agarose gels in 1× TBE buffer running at 65 V constant for 1.5–2 h and then stained by ethidium bromide (1 μg·mL^−1^) and photographed under UV light in a gel documentation system (Villber Lourmat, France)

### 4.3. Data Analysis

All clearly detectable ISSR product bands were scored as either presence (1), absence (0) or ambiguous (9) of each band for all accessions and the matrix of ISSRs data was assembled. All amplifications were repeated at least twice and only reproducible and well-defined bands were considered for analysis.

Polymorphism information content (PIC) was calculated using the formula PIC = 1 − ∑*pi*^2^, where *pi* is the frequency of the *i*th allele [33]. Jaccard’s similarity coefficient values for each pairwise comparison between accessions were calculated and a similarity coefficient matrix was constructed. This matrix was subjected to an un-weighted paired group method with arithmetic average (UPGMA) [34] to generate a dendrogram using software NTSYS-pc Version 2.02 [35]. The MXCOMP subroutine was used to calculate a cophenetic correlation coefficient between the similarity matrix and respective dendrogram derived matrix to measure suitability-of-fit [36]. Winboot software [37] was used for a bootstrap analysis with 1000 replicates to obtain the confidence of branches of the cluster tree. The frequency of occurrence of each marker in each genotype was computed, to render a matrix of 27 accessions by ISSR markers. These matrices were subjected to principal coordinate analysis (PCoA) to show the relationship between genotypes.

Observed number of alleles (Na), effective number of alleles (Ne), Nei’s gene diversity (He) [38] and Shannon’s information index (I) [39] were estimated for total genotypes using POPGENE software version 1.32 [40].

## 5. Conclusion

In summary, our results indicate that the level of polymorphism among bermudagrass accessions is appreciably high in Iran. The results of this study also suggest that the ISSR marker offers a powerful means to analyze the genetic diversity among accessions. Cluster analysis using the UPGMA method and Jaccard’s similarity coefficient (*r* = 0.828) grouped the accessions into six main clusters according to some degree to geographical origin, their chromosome number and some morphological characteristics. The enlargement of the number of primer pairs and accessions would have provided more useful information to assist the classification of the bermudograss germplasm.

## Figures and Tables

**Table 1:** List of the inter-simple sequence repeat (ISSR) primers used in the present study, polymorphism number, rates and polymorphism information content (PIC) values.

Primer name	Nucleotide sequence	Tm (°C)	No. of fragments amplified	No. of polymorphic fragments	Percentage of poymorphism	PIC value
ISSR-1	5′-(AC)8 G-3′	54/9	23	19	82.6	0.313
ISSR-2	5′-(AC)8T-3′	49/2	29	25	86.2	0.266
ISSR-3	5′-(AC)8C-3′	53/3	25	20	80	0.291
ISSR-4	5′-(AG)8C-3′	46/8	24	20	83.3	0.280
ISSR-5	5′-(AG)8T-3′	42/5	28	26	92.8	0.205
ISSR-6	5′-(GA)8C-3′	43/3	25	22	88	0.283
ISSR-7	5′-(GA)8T-3′	42/9	36	26	72.6	0.333
ISSR-8	5′-(CT)8G-3′	44/9	30	20	66.7	0.346
ISSR-11	5′-(AG)8GT-3′	47/1	40	27	67.5	0.333
ISSR-14	5′-(AC)8GG-3′	57	20	16	80	0.375
ISSR-15	5′-(AC)8GA-3′	53/7	25	20	80	0.433
RAMP-TAG	5′-T(AG)9-3′	56/1	29	24	82.7	0.428
RAMP-GAC	5′-G(AC)9-3′	53/9	25	22	88	0.391
LK7	5′-CCA(CT)8-3′	56/1	30	26	86.7	0.321
**Total means**	**-**	**-**	**389**	**313**	**80.5**	**0.328**

**Table 2:** List of the accessions of *Cynodon dactylon* (L.) Pers. used in the present study.

Accession name	Collected site (province-city)	Altitude (m)	Annual Rainfall (mm)	Main daily temperature (°C)	Choromosome No.
1-Lr	Charmahal Bakhtiari-Lordegan	1900	551	17/5	36
3-Gs	Charmahal Bakhtiari-Borujen	2200	245	12/8	36
6-Gs	Charmahal Bakhtiari-Borujen	1930	245	12/8	36
11-Ns	Isfahan-Natanz	1800	198	16/4	36
13-Ns	Isfahan-Natanz	1800	198	16/4	36
15-Bs	Isfahan-Natanz	1300	198	16/4	36
17-GN1	Mazandaran-Neka	15	895	16/8	18
18-Gr	Gilan-Chaboksar	15-	1220	15/9	36
19-Gcr	Gilan-Chaboksar	15-	1220	15/9	36
24-II	Isfahan-Isfahan	2060	122	16/2	36
27-II	Isfahan-Isfahan	1577	122	16/2	36
36-Tr	Isfahan-Isfahan	1580	122	16/2	36
39-II	Isfahan-Isfahan	1573	122	16/2	36
44-II	Isfahan-Isfahan	1570	122	16/2	27
51-Chg	Isfahan-Chadegan	2150	317	10/2	36
52-Chg	Isfahan-Chadegan	2150	317	10/2	36
53-Chr	Isfahan-Chadegan	2150	317	10/2	36
55-Chg	Isfahan-Chadegan	2180	317	10/2	36
56-Chr	Isfahan-Chadegan	2180	317	10/2	36
58-Chr	Isfahan-Chadegan	2170	317	10/2	36
59-Chf	Isfahan-Chadegan	2160	317	10/2	36
78-Ts	Isfahan-Tiran	1900	175	14/5	36
88-Khl	Isfahan-Khomeini shahr	1560	130	15/8	18

## References

[1] Harlan J.R. (1970). *Cynodon* species and their value for grazing or hay. Herb. Abstr.

[2] Harlan J.R., de Wet J.M.J. (1969). Sources of variation in *Cynodon dactylon* (L.) Pers. Crop Sci.

[3] Gupta P.K., Varshney R.K. (2000). The development and use of microsatellite markers for genetic analysis and plant breeding with emphasis on bread wheat. Euphytica.

[4] Reddy M.P., Sarla N., Siddiq E.A. (2002). Inter simple sequence repeat (ISSR) polymorphism and its application in plant breeding. Euphytica.

[5] Zietkiewicz E., Rafalski A., Labuda D. (1994). Genome fingerprinting by simple sequence repeat (SSR)-anchored polymerase chain reaction amplification. Genomics.

[6] Barth S., Melchinger A.E., Lubberstedt T.H. (2002). Genetic diversity in *Arabidopsis thaliana* L. Heynh. Investigated by cleaved amplified polymorphic sequence (CAPS) and intersimple sequence repeat (ISSR) markers. Mol. Ecol.

[7] Huang C.Q., Liu G.D., Bai C.J., Wang W.Q., Zhou S.Y., Yu D.Q. (2010). Estimation of genetic variation in *Cynodon dactylon* accessions using the ISSR technique. Biochem. Syst. Ecol.

[8] Reunova G.D., Kats I.L., Muzarok T.I., Zhuravleu I. (2010). Polymerase of RAPD, ISSR and AFLP markers of the *Panax ginseng* C.A. Meyer (Araliaceae) genome. Genetika.

[9] Smolik M., Ochmian I., Grajkowski J. (2010). Genetic variability of Polish and Russian accessions of cultivated blue honeysuckle (*Lonicera caerulea*). Genetika.

[10] Zehdi S., Trifi M., Salem A., Rhouma A., Marrakchi M. (2002). Survey of inter simple sequence repeat polymorphisms in Tunisian date palms (*Phoenix dactylifera* L.). J. Genet. Breed.

[11] Gulsen O., Sever S., Mutlu N., Tuna M., Karaquzel O., Shearman R.C. (2009). Ployploidy creates higher diversity among *Cynodon* accessions as assessed by molecular markers. Theor. Appl. Genet.

[12] Arcade A., Anselin F., Faivye P., Lesage M.C., Paques L.E., Prat D. (2004). Application of AFLP, RAPD and ISSR markers to genetic mapping of European and Japanese larch. Theor. Appl. Genet.

[13] Budak H., Shearman R.C., Gulsen O., Dweikat I. (2005). Understanding ploidy complex and geographic origin of the *Buchloe dactyloides* genome using cytoplasmic and nuclear marker systems. Theor. Appl. Genet.

[14] Ge X.J., Yu Y., Yuan Y., Huang H.W., Yan C. (2005). Genetic diversity and geographic differentiation in endangered *Ammopiptanthus* (Leguminosae) populations in desert regions of northwest China as revealed by ISSR analysis. Ann. Bot.

[15] Raina S.N., Rani V., Kojima T., Ogihara Y., Singh K.P., Devarumath R.M. (2001). RAPD and ISSR fingerprints as useful genetic markers for analysis of genetic diversity, varietal identification, and phylogenetic relationships in peanut (*Arachis hypogaea*) cultivars and wild species. Genome.

[16] Zhang L., Li Q.J., Li H.T., Chen J., Li D.Z. (2006). Genetic diversity and geographic differentiation in *Tacca chantrieri* (Taccaceae) san autonomous selfing plant with showy floral display. Ann. Bot.

[17] Anderson M.P., Taliaferro C.M., Martin D.L., Anderson C.S. (2001). Comparative DNA profiling of U-3 turf bermudagrass strains. Crop Sci.

[18] Etemadi N., Sayed-tabatabaei B.E., Zamani Z., Razmjoo K.H., Khalighi A., Lessani H. (2006). Evaluation of diversity among *Cynodon dactylon* (L.) Pers. using RAPD markers. Agric. Biol.

[19] Roodt R., Spies J.J., Burger T.H. (2002). Preliminary DNA fingerprinting of the turf grass *Cynodon dactylon* (Poaceae: Chloridoideae). Bothalia.

[20] Karaca M., Saha S., Zipf A., Jenkins J.N., Lang D.J. (2002). Genetic diversity among bermudagrass (*Cynodon* spp.): Evidence from chloroplast and nuclear DNA fingerprinting. Crop Sci.

[21] Wu Y., Taliaferro C.M., Bai G.H., Anderson M.P. (2004). AFLP analysis of *Cynodon dactylon* (L.) Pers. Var. dactylon genetic variation. Genome.

[22] Li H., Liu L., Lou Y., Hu T., Fu J. (2011). Genetic diversity of Chinese natural bermudagrass (*Cynodon dactylon*) germplasm using ISSR markers. Sci. Hortic.

[23] Kamps T.L., Williams N.R., Ortega V.M., Chamusco K.C., Harris-Shultz K., Scully B.T., Chase C.D. (2011). DNA polymorphisms at bermudagrass microsatellite loci and their use in genotype fingerprinting. Crop Sci.

[24] Vaiman D., Mercier D., Moazami-Goudarzi K., Eggen A., Ciampolini R., Lépingle A., Velmala R., Kaukinen J., Varvio S.L., Martin P., Levéziel H., Guérin G. (1994). A set of 99 cattle microsatellite, characterization, synteny mapping and polymorphism. Mamm. Genome.

[25] Xie W., Zhang X., Cai H., Liu W., Peng Y. (2010). Genetic diversity analysis and transferability of cereal EST-SSR markers to orchardgrass (*Dactylis glomerata* L.). Biochem. Syst. Ecol.

[26] Tajima F., Tokunaga T., Miyashita N.T. (1994). Statistical methods for estimating the effective number of alleles, expected heterozygosity and genetic distance in self-incompatibility locus. Jpn. J. Genet.

[27] Mohammadi S.A., Prasanna B.M. (2003). Analysis of genetic diversity in crop plants—Salient statistical tools and considerations. Crop Sci.

[28] Burton G.W., Janick J., Simon J.E. (1993). African Grasses.

[29] Zhang L.H., Ozias-Akins P., Kochert G., Kresovich S., Dean R., Hanna W. (1999). Differentiation of bermudagrass (*Cynodon* spp.) genotypes by AFLP analyses. Theor. Appl. Genet.

[30] Caetano-Anolles G. (1998). DNA analysis of turfgrass genetic diversity. Crop Sci.

[31] Yi Y., Zhang X., Huang L., Ling Y., Ma X., Liu W. (2008). Genetic diversity of wild *Cynodon dactylon* germplasm detected by SRAP markers. Yi Chuan.

[32] Dellaporta S.L., Wood J., Hicks J.B. (1983). A plant DNA minipreparation: Version II. Plant Mol. Biol. Rep.

[33] Smith J.S.C., Chin E.C.L., Shu H., Smith O.S., Wall S.J., Senior M.L., Mitchell S.E., Kresovich S., Zeigle J. (1997). An evaluation of the utility of SSR loci as molecular markers in maize (*Zea mays* L.): Comparison with data from RFLPs and pedigree. Theor. Appl. Genet.

[34] Michener C.D., Sokal R.R. (1957). A quantitative approach to a problem of classification. Evolution.

[35] Rohlf F.J. (1998). *NTSYSpc. Numerical Taxonomy and Multivariate Analysis System*, version 2.02; Exeter software.

[36] Sokal R.R., Rohlf F.J. (1962). The comparison of dendrograms by objective methods. Taxon.

[37] Yap I.V., Nelson R.J. (1996). Winboot: A Program for Performing Bootstrap Analysis of Binary Data to Determine the Confidence Limits of UPGMA-based Dendrograms.

[38] Nei M. (1978). Estimation of average heterozygosity and genetic distance from a small number of individuals. Genetics.

[39] Shannon C.E., Weaver W. (1949). The Mathematical Theory of Communication.

[40] Yeh F.C., Yang R.C., Boyle T., Ye Z.H., Mao J.X. (1999). POPGENE, the User Friendly Shareware for Population Genetic Analysis.

